# Direct Affinity Ligand Immobilization onto Bare Iron Oxide Nanoparticles Enables Efficient Magnetic Separation of Antibodies

**DOI:** 10.1021/acsabm.4c00280

**Published:** 2024-05-13

**Authors:** Ines Zimmermann, Yasmin Kaveh-Baghbaderani, Friederike Eilts, Nadja Kohn, Paula Fraga-García, Sonja Berensmeier

**Affiliations:** † Chair of Bioseparation Engineering, TUM School of Engineering and Design, Technical University of Munich, Boltzmannstraße 15, 85748 Garching, Germany; ‡ Munich Institute of Integrated Materials, Energy and Process Engineering, Technical University of Munich, Lichtenbergstraße 4a, 85748 Garching, Germany

**Keywords:** magnetic nanoparticles, downstream processing, site-directed Protein A immobilization, pH buffering of iron oxides, protein recovery, kinetics

## Abstract

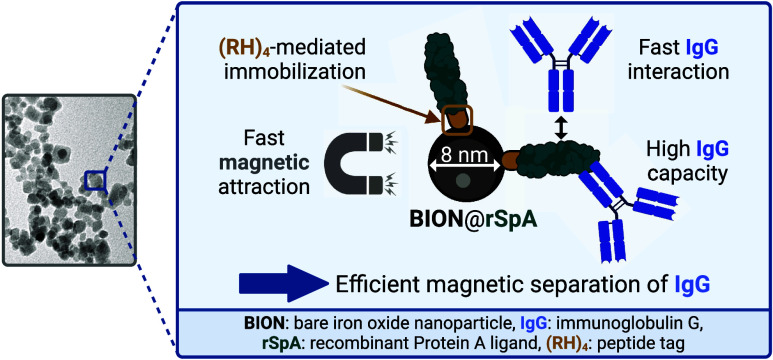

Magnetic separation is a promising alternative to chromatography for enhancing the downstream processing (DSP) of monoclonal antibodies (mAbs). However, there is a lack of efficient magnetic particles for successful application. Aiming to fill this gap, we demonstrate the suitability of bare iron oxide nanoparticles (BION) with physical site-directed immobilization of an engineered Protein A affinity ligand (rSpA) as an innovative magnetic material. The rSpA ligand contains a short peptide tag that enables the direct and stable immobilization onto the uncoated BION surface without commonly required laborious particle activation. The resulting BION@rSpA have beneficial characteristics outperforming conventional Protein A-functionalized magnetic particles: a simple, fast, low-cost synthesis, a particle size in the nanometer range with a large effective specific surface area enabling large immunoglobulin G (IgG) binding capacity, and a high magnetophoretic velocity advantageous for fast processing. We further show rapid interactions of IgG with the easily accessible rSpA ligands. The binding of IgG to BION@rSpA is thereby highly selective and not impeded by impurity molecules in perfusion cell culture supernatant. Regarding the subsequent acidic IgG elution from BION@rSpA@IgG, we observed a hampering pH increase caused by the protonation of large iron oxide surfaces after concentrating the particles in 100 mM sodium acetate buffer. However, the pH can be stabilized by adding 50 mM glycine to the elution buffer, resulting in recoveries above 85% even at high particle concentrations. Our work shows that BION@rSpA enable efficient magnetic mAb separation and could help to overcome emerging bottlenecks in DSP.

## 1 Introduction

Monoclonal antibodies (mAbs) are essential biopharmaceuticals for treating numerous diseases. The market demand and annual mAb approvals increase continuously.^1−3^ Facing the challenge of efficiently producing large amounts of mAbs, multiple upstream processing (USP) advances have resulted in enhanced expression productivity over the last decades.^4,5^ However, the subsequent downstream processing (DSP) cannot keep pace with the progress in USP, essentially caused by the predominating packed-bed Protein A chromatography capture step.^6,7^ Although Protein A ligands are suitable as they allow selective binding to various mAb types,^8,9^ diffusional mass transfer constrains the throughput and the productivity in the packed-bed chromatography operation.^10,11^ Furthermore, there are capacity and scalability limitations.^12,13^ Despite excellent yields and purities achieved with Protein A chromatography, efficient alternative capture operations are thus needed and of particular research focus.^6,14−19^

We consider magnetic separation a highly promising alternative, which has found increasing popularity in recent years.^15−17,20−24^ This technique is based on nonporous magnetic particles that are freely dispersed in the process fluid and can be controlled magnetically. The nonporous magnetic particle adsorbent leads to reduced mass transfer limitations compared to conventional porous chromatography beds, which favors fast target adsorption/desorption and, thus, high throughput.^25,26^ Furthermore, clogging of the purification matrix is prevented due to the nonporosity, enabling the direct processing of unclarified cell culture broth and process intensification.^16,19^ Another powerful characteristic of the technique is the scale-independence of the magnetic adsorption process, which simplifies the scale-up.^27^

Despite the stated advantages, no large-scale industrial magnetic separation application has been installed for mAb DSP. The market introduction of a current good manufacturing practice (cGMP)-compliant rotor-stator high-gradient magnetic separator (RS-HGMS) in 2017 marks an important milestone for accelerated research on pilot-scale magnetic mAb separation.^28^ However, we have identified conventional Protein A-functionalized magnetic particles as an essential bottleneck of current research studies. Three major drawbacks are (i) a particle size in the micrometer range that has a lower specific surface area for protein binding compared to nanoparticles; (ii) the use of non-oriented affinity ligand immobilization, known to further reduce the mAb binding capacity,^29,30^ and (iii) expensive, labor-intensive particle modifications (e.g., coatings), which usually decrease the magnetization^31,32^ and the specific surface area for protein interactions.^32,33^

To counteract the described bottleneck of conventional particles, Kaveh–Baghbaderani et al. have developed cheap and simply synthesized bare iron oxide magnetic nanoparticles (BION) with site-directed immobilization of an engineered Protein A-based affinity ligand (rSpA).^34^ The rSpA ligand comprises eight B-domains of Protein A and a fused arginine-histidine tag ((RH)_4_). The latter enables the direct ligand immobilization on the BION surface via coordinative and ionic bonding. In contrast to conventional ligand immobilization approaches, no chemical BION modification is thus required. In a promising proof-of-concept study on adsorption and desorption isotherms of polyclonal IgG, Kaveh–Baghbaderani et al. already demonstrated high binding capacities.^34^

In the present work, we took the characterization of the BION@rSpA particles one step further toward the process applicability for a monoclonal IgG. We first compared process-relevant particle characteristics resulting from the direct rSpA immobilization versus conventional immobilization strategies. We analyzed particle sizes by transmission electron microscopy (TEM) and agglomeration by dynamic light scattering (DLS) and ζ-potential measurements. In addition, we investigated the magnetic behavior with a superconducting quantum interference device (SQUID) and space- and time-resolved extinction profiles (STEP). Furthermore, we examined the process stability of the rSpA immobilization onto the BION. Regarding IgG interactions, we investigated kinetics, the impact of impurities in clarified cell culture supernatant on IgG adsorption, the influence of mixing speeds, and the IgG desorption at varying particle concentrations. The studies provide fundamental insights into magnetic separation based on uncoated iron oxide nanoparticles, which enable us to evaluate the process applicability of BION@rSpA for the purification of monoclonal IgG with an eye on future large-scale process development.

## 2 Experimental Section

### 2.1 Particle Synthesis

#### 2.1.1 BION and BION@rSpA

Bare iron oxide nanoparticles (BION) were synthesized via coprecipitation^35^ following the general procedure by Roth et al.^36^ In short, 86.4 g FeCl_3_·6H_2_O and 35.0 g FeCl_2_·4H_2_O were dissolved in 400 mL deionized and degassed water and added dropwise to 1 L of 1.8 M NaOH. After stirring for 30 min, the iron oxide particles were magnetically separated and washed with deionized, degassed water until the conductivity was below 200 μS cm^–1^.

The rSpA affinity ligand was expressed in *Escherichia coli* BL21 (DE3) containing a pET28a plasmid with the coding gene and subsequently purified via the histidine-containing tag using immobilized metal affinity chromatography (IMAC) as described by Kaveh–Baghbaderani et al.^34^

Ligand immobilization onto BION can be done at different particle concentrations when keeping a consistent ligand-to-particle ratio. The immobilization was done in low-binding tubes (Protein LoBind; Eppendorf, Germany) by simple incubation of BION (1–5 g L^–1^) with rSpA ligand in 20 mM Tris, 150 mM NaCl at pH 7 (Tris-buffered saline (TBS)). If not stated differently, 0.15 g g^–1^ rSpA was used, and incubation conditions were 1000 rpm, 25 °C for 1 h (ThermoMixer C; Eppendorf, Germany). After the functionalization in TBS, the BION@rSpA were incubated in all the process buffers used for IgG separation (adsorption, wash, and elution buffers) for at least 30 min to ensure stable ligand immobilization throughout the process. PBS buffer (20 mM KH_2_PO_4_, 150 mM NaCl at pH 7.4) was used to imitate cell culture supernatant. Between the incubation steps, the particles were washed twice with the previous buffer, followed by one wash step with deionized water. After the functionalization procedure, the BION@rSpA particles were rebuffered into TBS and stored at 4 °C until further use.

#### 2.1.2 ION@TEOS

We compared the particle characteristics of BION and BION@rSpA to iron oxide nanoparticles (ION) coated with tetraethyl orthosilicate (TEOS, >99%; Merck, Germany). A synthesis protocol by Turrina et al. was followed with a molar equivalent of 1.96 TEOS to reach a complete silica coating of the iron oxide surface.^32^

### 2.2 Particle Characterization

Analytical methods were done at room temperature if not stated differently. Particle concentrations were determined via dry mass analysis (dried for 3 days at 80 °C) and a phenanthroline assay as orthogonal methods. The detailed procedure followed for the phenanthroline assay can be found in Supporting Information (S.1).

In preparation for transmission electron microscopy (TEM), the particles were suspended in water at a concentration of 0.025 g L^–1^ and sonicated for 30 min. Afterward, 30 μL of the suspension was applied onto a carbon grid (300 mesh, copper; Micro to Nano, Netherlands). The grid was dried with heated air, and the preparation was then inspected using a TEM JEM 1400 Plus device (Jeol, Japan). Average particle sizes were determined by evaluating 100 particles using ImageJ.^37^

A superconducting quantum interference device (SQUID) magnetometer MPMS XL-7 (Quantum Design, Germany) was used to determine the magnetization of the particles. Here, a defined amount of particles (10 mg) was glued onto a small plastic tube (Fixogum; Marabu, Germany), and the sample was then analyzed in a varying magnetic field (±50 kOe).

Hydrodynamic diameters and ζ-potentials were analyzed in TBS (20 mM Tris, 150 mM NaCl, pH 7.0) and sodium acetate (100 mM, pH 2.8) as exemplary process buffers. The hydrodynamic diameters were analyzed by dynamic light scattering (DLS) using a Zetasizer Ultra system (Malvern Panalytical, U.K.). The same device was used for the determination of ζ potentials. For both measurements, particle solutions (1 g L^–1^) were homogenized in an ultrasonic bath for 3 min before the measurement of 1 mL in the respective cuvettes for DLS (macro cuvette PS; VWR) or ζ potential (cell DTS1070; Malvern Panalytical, U.K.). Measurements were conducted and evaluated with the device-related ZS Xplorer software using predefined parameters for magnetite (refractive index: 2.36, absorption: 0.147).

Space- and time-resolved extinction profiles (STEP) of particles (1 g L^–1^) were analyzed in the same exemplary process buffers used for DLS and ζ measurements. A LUMiReader (LUM GmbH, Germany) modified with five stacked cylindrical neodymium boron ferrite (NdFeB) magnets (Webcraft GmbH, Germany) was used. The corresponding magnetic field strength above the magnets can be found in the Supporting Information (Figure S.1). The samples (1 mL) were measured in cuvettes (macro cuvette PS; VWR), and extinction profiles at 870 nm were taken to evaluate the magnetophoresis (SEPView software; LUM GmbH, Germany).

### 2.3 Protein Analysis

A bicinchoninic acid (BCA) protein assay kit (Pierce; Thermo Fisher Scientific) was used for ligand quantification, following the instructions given by the manufacturer. For rSpA quantification, a standard curve of recombinant Protein A (Sino Biological, China) was prepared in the respective buffer. A modified particle BCA assay was performed to quantify the ligand load on particles as described previously.^34^ BION of the respective concentration were thereby used for setting the zero value.

Pure antibody samples were quantitatively analyzed using a NanoPhotometer (Implen, Germany). The respective buffer was used to set the blank value, and the default parameter for human IgG was used for the concentration analysis.

For the quantification of antibodies in cell culture supernatant, Protein A (UNOsphere SUPrA Resin; Bio-Rad, Germany) high-performance liquid chromatography (HPLC; 1260 Infinity II; Agilent) was used with 0.02 M NaH_2_PO_4_, 0.02 M sodium citrate at pH 7.5 as equilibration and 0.02 M sodium citrate, and 0.1 M sodium chloride at pH 2.9 as elution buffer. A calibration standard of pure IgG measured with the NanoPhotometer was used.

Protein profiles were qualitatively investigated using sodium-dodecyl sulfate-polyacrylamide gel electrophoresis (SDS-PAGE). Antibody-containing samples were heated in a loading buffer for 5 min at 95 °C under nonreducing conditions. Samples were run on a 12% acrylamide gel in Tris-Glycine buffer (AppliChem, Germany). Scanned gels (Amersham Typhoon; Cytiva) were analyzed with the corresponding ImageQuant TL software (version 8.2; Cytiva).

DLS (Zetasizer Ultra; Malvern Panalytical, U.K.) was used to estimate antibody agglomeration in elution fractions. Elution samples were neutralized with 1 M Tris (pH 8) buffer before the measurement. For the measurement, the parameters for standard proteins predefined in the related ZS Xplorer Software were used (refractive index: 1.45; absorption: 0.001). Samples (200 μL) were measured in microcuvettes (Sarstedt, Germany).

### 2.4 IgG Adsorption and Desorption Using BION@rSpA

In IgG interaction studies, an IgG1 monoclonal antibody was used in a purified form in TBS buffer (20 mM Tris, 150 mM NaCl at pH 7.0) or in perfusion cell culture supernatant, which was kindly received from Bilfinger SE (Vienna, Austria) and the Department of Industrial Biotechnology at KTH Royal Institute of Technology (Stockholm, Sweden). The synthesized BION@rSpA were used for IgG capture after equilibration in TBS buffer. If not stated otherwise, incubation of particles with IgG was done at 1000 rpm, 25 °C for 1 h (ThermoMixer C; Eppendorf, Germany). Depending on the experiment, the concentrations ranged from 1–20 g L^–1^ BION@rSpA and 0.10–0.40 g IgG per g particles. After IgG adsorption, three wash steps were performed using TBS buffer as described by Kaveh–Baghbaderani et al.^34^ For elution, the particles were transferred to an elution buffer and incubated for 1 h at 1000 rpm, 25 °C, unless indicated otherwise. Elution buffers containing sodium acetate (0, 50, 100, or 200 mM), glycine (0 or 50 mM), and sodium chloride (0 or 150 mM) were tested at different pH values between 2.4 and 3.7.

## 3 Results and Discussion

### 3.1 Characteristics of BION, BION@rSpA, and ION@TEOS

The focus of particle characterization was on the size (TEM), agglomeration (DLS and ζ-potential), and magnetic behavior (SQUID and STEP) as relevant parameters for process productivity. In addition to BION and ligand-functionalized BION@rSpA, we characterized silica-coated iron oxide nanoparticles (ION@TEOS) to compare the effects of a typical particle modification and the sole rSpA immobilization. The silica coating was chosen as an example because of its wide use in covalent ligand immobilization protocols.^29,38−40^

**Figure 1:**
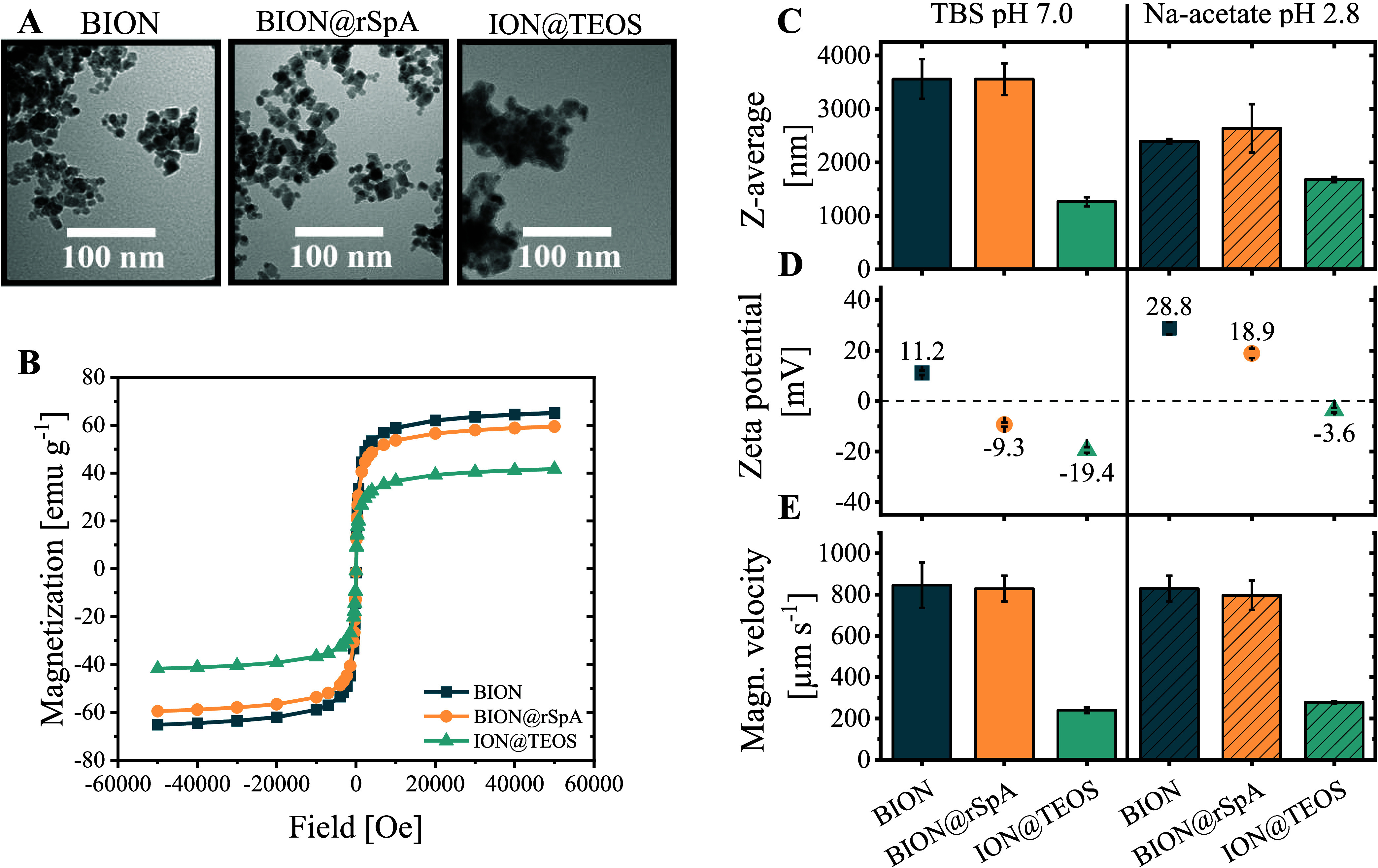
Characteristics of BION, BION@rSpA, and ION@TEOS. (A) TEM images of the particles. Determined particle sizes are *d*_BION_: 8.35 ± 1.40 nm, *d*_BION@rSpA_: 8.44 ± 1.46 nm, *d*_ION@TEOS_: 12.00 ± 0.13  nm. (B) SQUID measurements. (C) Hydrodynamic diameters as *z*-average values analyzed by DLS in TBS (20 mM Tris, 150 mM NaCl, pH 7.0) and sodium acetate (100 mM, pH 2.8). The same buffers were used for (D) ζ-potential measurements and (E) space-and time-resolved extinction profiles (870  nm) for magnetophoretic velocity determinations. Error bars represent the standard deviation of three individual measurements. Data from ION@TEOS plotted in (A) and (B) were reproduced from Turrina et al.^32^

The analysis of TEM images revealed no noticeable difference between the average particle sizes of BION (*d*_BION_: 8.35 ± 1.40 nm) and BION@rSpA (*d*_BION@rSpA_: 8.44 ± 1.46 nm) (Figure 1A). In comparison, ION@TEOS possessed a visible silica coating and a larger average diameter (*d*_ION@TEOS_: 12.00 ± 0.13 nm). BION and BION@rSpA magnetization measurements revealed similar maximum values of 65 and 60 emu g^–1^, whereas 42 emu g^–1^ was determined for ION@TEOS (Figure 1B). *Z*-average values measured by DLS in the exemplary process buffers TBS (BION: 3562.7 ± 372.0 nm; BION@rSpA: 3560.7 ± 296.2 nm; ION@TEOS: 1267.3 ± 85.7 nm) and 100 mM sodium acetate at pH 2.8 (BION: 2397.0 ± 45.3 nm; BION@rSpA: 2640.3 ± 451.8 nm; ION@TEOS: 1681.3 ± 47.1 nm) were larger by several orders of magnitude than the core sizes analyzed by TEM (Figure 1C). Again, BION and BION@rSpA showed similar behavior, differentiating from ION@TEOS particles. Determined ζ-potentials of all investigated particles were in general more positive in 100 mM sodium acetate at pH 2.8 (BION: 28.8 ± 2.5 mV; BION@rSpA: 18.9 ± 1.9 mV; ION@TEOS: −3.6 ± 0.9 mV) than in TBS buffer at pH 7.0 (BION: 11.2 ± 0.9 mV; BION@rSpA: −9.3 ± 0.8 mV; ION@TEOS: −19.4 ± 1.2 mV) (Figure 1D). Regarding the magnetophoretic attraction, determined velocities of BION and BION@rSpA were comparable in both investigated process buffers (600–800 μm s^–1^). In contrast, the ION@TEOS showed slower magnetophoretic attraction in the used measuring device setup (around 240 μm s^–1^) (Figure 1E).

All analytical methods revealed similar behavior between BION and BION@rSpA that distinguished from the coated ION@TEOS. As expected, the coating of ION@TEOS increased the core particle size (Figure 1A). The increased particle size can disadvantage bioseparation applications as the specific surface area decreases. For example, Turrina et al. measured a specific surface area of BION (103.0 m^2^ g^–1^) twice that of the coated ION@TEOS (50.6 m^2^ g^–1^).^32^ It thus becomes apparent that the uncoated particles, which form the basis of BION@rSpA, can provide a larger specific surface area for ligand and IgG adsorption than coated particles, which are the basis for common covalent ligand immobilization strategies. We could not find any reports of smaller Protein A-functionalized particles than BION@rSpA in the literature or on the commercial market. The small size with a large specific surface area likely contributes to the high recovery of polyclonal IgG from BION@rSpA (0.42 g_IgG_ g_BION@rSpA_), which exceeds the state-of-the-art as previously reported by Kaveh–Baghbaderani et al.^34^

Larger hydrodynamic diameters of the particles determined by DLS compared to the core sizes determined by TEM arise from particle agglomeration in solution. BION mainly agglomerate due to lacking electrostatic repulsion. Thus, the tendency of particle agglomeration is usually the highest at the isoelectric point (pI), where the ζ-potential is zero.^41,42^ In contrast, it decreases with larger absolute ζ-potentials due to electrostatic repulsion. We observed similar agglomeration of BION@rSpA and BION in TBS buffer, whereas slightly higher BION@rSpA agglomeration was seen in sodium acetate buffer (Figure 1C). In TBS buffer, BION and BION@rSpA showed similar absolute ζ-potentials despite a shift from positive to negative due to the rSpA immobilization (Figure 1D), which agrees with the similar agglomeration behavior observed. In sodium acetate, the absolute ζ- potential of BION was around 10 mV larger than that of BION@rSpA, resulting in more electrostatic repulsion between the BION and, thus, probably less agglomeration. Compared to TBS, the generally reduced agglomeration seen for BION and BION@rSpA in sodium acetate also aligns with the higher absolute ζ-potential values.

Based on the DLS measurements, agglomeration was reduced for ION@TEOS compared to the uncoated particles in both investigated process buffers (Figure 1C). The low electrostatic repulsion assumed from the smallest absolute ζ-potential value of ION@TEOS in sodium acetate buffer (−3.6 ± 0.9 mV; Figure 1D) did not lead to the highest agglomeration. This stabilizing effect of TEOS and various other coatings is known.^32,43^ The reduction of magnetic dipole interactions due to shielding by the TEOS coating could have contributed to the lower agglomeration.^44^

The determined magnetization of BION and the only slight decrease by around 5 emu g^–1^ upon protein adsorption are consistent with already published data.^45,46^ Also, the strong shielding of the magnetization due to the coating of ION@TEOS agrees with the literature.^38,47^ The larger intrinsic magnetization of BION and BION@rSpA likely contributed to the generally faster determined magnetophoretic sedimentation velocities compared to ION@TEOS (Figure 1E). In addition, the magnetophoretic velocity strongly correlates with the hydrodynamic diameter.^41,48^ Thus, the increased agglomeration seen for the uncoated particles probably also benefited the fast magnetophoretic attraction.

Regarding ligand adsorption into the agglomerates, we believe that rSpA diffuses into the loosely packed agglomerates as we did not notice varying ligand loadings on BION with different agglomeration states throughout our work. The literature also indicates the highly accessible BION surface despite agglomeration.^49,50^

In summary, the particle characterization demonstrated that the silica coating, often used in conventional ligand immobilization strategies, has a greater impact on the advantageous intrinsic properties of BION (small size, fast magnetophoretic attraction) than the actual immobilization of ligands.

### 3.2 IgG Adsorption and Desorption

#### 3.2.1 Kinetics

IgG adsorption and desorption kinetics significantly influence the required process time and, thus, the productivity of a separation process.^10^ In our studies, we conducted adsorption experiments with two initial IgG concentrations (0.10–0.12 g_IgG_ g_BION@rSpA_^–1^ and 0.40 g_IgG_ g_BION@rSpA_^–1^) to examine the impact of under- and oversaturated ligand binding sites. Furthermore, we investigated pure IgG in TBS buffer and IgG in perfusion cell culture supernatant to see the impact of impurity molecules on adsorption. The titer in the cell culture supernatant was 0.4 g L^–1^ IgG; thus, 1 or 3 g L^–1^ BION@rSpA were applied to reach the oversaturated and undersaturated approaches.

**Figure 2:**
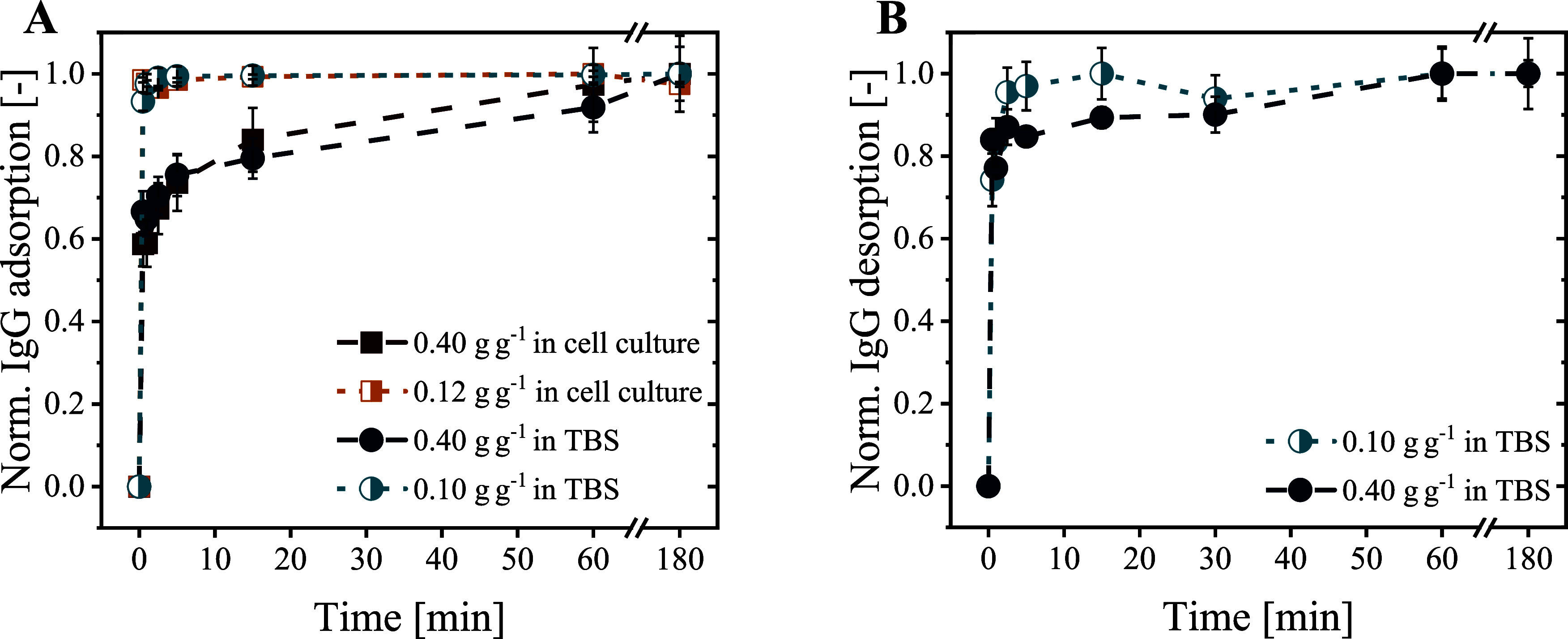
(A) IgG adsorption and (B) desorption kinetics onto/from BION@rSpA are shown. Adsorption in (A) was studied with pure IgG in TBS buffer (20 mM Tris, 150 mM NaCl, pH 7.0) and IgG in cell culture supernatantat at two initial IgG concentrations (0.10–0.12 and 0.40 g g^–1^). For desorption in (B), the same two initial IgG concentrations were considered for IgG binding in TBS buffer (60 min) before desorption in 100 mM sodium acetate and 50 mM glycine at pH 2.8. Values were normalized to the maximum value within 180 min of adsorption/desorption time.

As visualized in Figure 2A, over 90% of the maximum bound IgG (∼0.10 g_IgG_ g_BION@rSpA_^–1^) was adsorbed after 30 s in the undersaturated approaches, while it took over 30 min to reach 90% (∼0.25 g_IgG_ g_BION@rSpA_^–1^) in the oversaturated approaches. However, the absolute bound IgG after 30 s was higher in the oversaturated (∼0.17 g_IgG_ g_BION@rSpA_^–1^) than in the lower concentrated approaches (∼0.10 g_IgG_ g_BION@rSpA_^–1^), driven by the larger antibody concentration gradient between the bulk phase and the particle’s surface (Figure S.2).^11,51^ The faster relative adsorption observed at lower initial IgG concentration likely resulted from the excess of binding sites, as the probability of IgG adsorption increases with the availability of binding sites.^52^ The larger IgG occupancy resulting from the higher initial IgG concentration probably sterically hindered the accessibility of binding sites for further IgG molecules.

In a study using Protein A Mag Sepharose magnetic microparticles (GE Healthcare), Ebeler et al. observed over 90% of the maximum IgG binding after 7–10 min.^15^ As they worked below the maximum IgG binding capacity, our results indicate faster IgG binding to BION@rSpA than to the commercial Protein A-functionalized magnetic particles. Furthermore, we observed faster IgG adsorption to BION@rSpA than Cao et al. to magnetic cellulose microspheres functionalized with Protein A.^53^ The high affinity of IgG molecules to the eight polymerized B-domains in rSpA and the efficient accessibility through the peptide tag-mediated, oriented ligand immobilization probably contributed to the quick IgG binding. Compared to chromatographic bead material, we also observed faster IgG adsorption to our BION@rSpA, likely due to the more efficient availability of binding sites resulting from the general nonporosity of the magnetic particles in contrast to the porous chromatographic material.^51^

No notable interference was detected concerning the influence of impurity molecules in the cell culture supernatant competing for IgG binding sites. First, both purified IgG and cell culture supernatant containing IgG showed the same maximum IgG binding (0.29 g g_BION@rSpA_^–1^) on BION@rSpA (Figure S.2). And second, selective IgG adsorption was seen in SDS-PAGE analysis (Figure S.3). This confirms the selective interaction between the ligand’s B domains with IgG.

As was observed for adsorption, acidic desorption of IgG from BION@rSpA@IgG also revealed rapid kinetics (Figure 2B). Again, desorption was faster after IgG adsorption at the lower initial concentration than at the higher IgG concentration. Over 90% of the desorbed IgG eluted within the first 2 and 15 min, respectively. It can be assumed that the elution buffer reached all the binding sites within a short mixing time, and the desorption of IgG from the ligands was thus not noticeably hindered by other bound IgG molecules. IgG molecules that were adsorbed on not directly accessible ligand sites in particle agglomerates rather contributed to the slower desorption in the samples incubated with excess IgG.

#### 3.2.2 Stability of rSpA Immobilization in Elution Buffers

**Figure 3:**
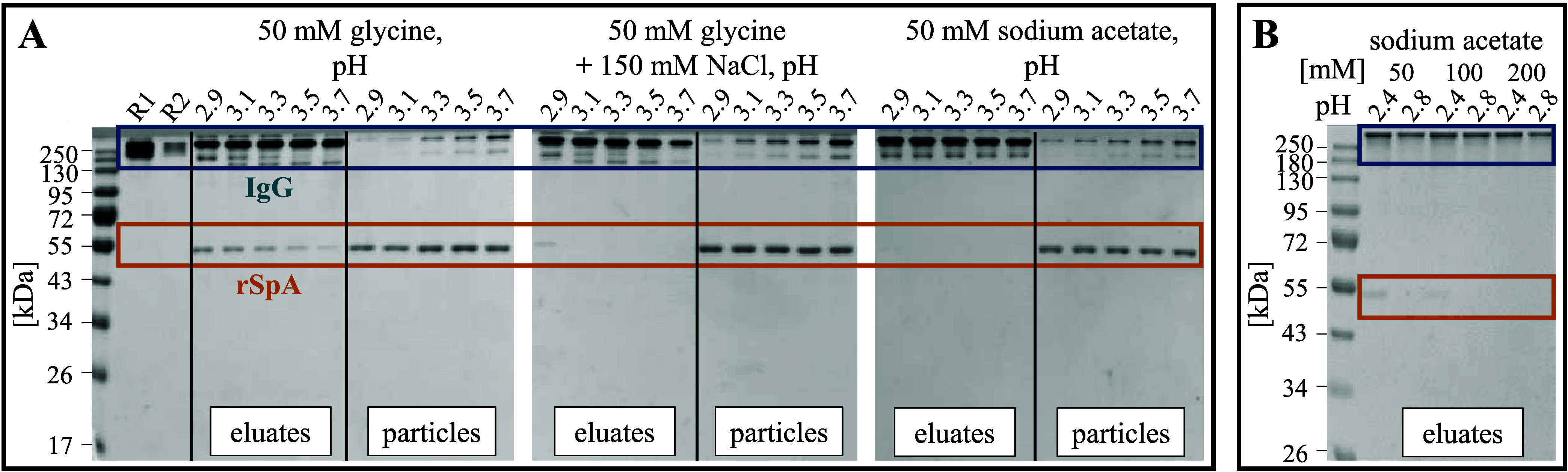
Investigation of rSpA immobilization stability in different elution buffers. Eluted IgG (“eluates”) and used particles after elution (“particles”) were analyzed in nonreducing SDS-PAGE. Gels were evaluated for rSpA leaching using the software ImageQuant TL (Cytiva). (A) Elution was investigated in 50 mM glycine, 50 mM glycine + 150 mM NaCl, and 50 mM sodium acetate at pH values between 2.9 and 3.7. A BION@rSpA concentration of 1.5 g L^–1^ was used. IgG reference samples of (R1) 0.500 g L^–1^ and (R2) 0.075 g L^–1^ are also shown. (B) Sodium acetate concentrations varied from 50 to 200 mM at pH values of 2.4 and 2.8. Here, 1.0 g L^–1^ BION@rSpA was used.

Stable rSpA immobilization on the BION surface is necessary to ensure the reusability of the particles and to prevent potential contamination of the mAb product with leaked ligand. In an elution buffer screening, we investigated the immobilization stability of rSpA in glycine, sodium chloride, and sodium acetate at pH values ranging from 2.9 to 3.7. In general, rSpA leaching (∼55 kDa) into the eluates increased with decreasing pH, as seen in SDS-PAGE analytics in Figure 3A. Leaching was evident at all pH values in 50 mM glycine buffer. In contrast, sodium chloride addition (150 mM) stabilized the ligand immobilization as leaching was only visible at pH 2.9, and no further bands were detectable with the software ImageQuant TL. In 50 mM sodium acetate, slight leaching appeared at pH 2.9. We concluded that glycine destabilized the ligand immobilization, whereas sodium chloride and sodium acetate promoted the stabilization.

A plausible explanation for the ligand leaching observed in pure glycine buffer is the possible ionic surface coordination through electrostatic interaction between the negatively charged carboxy group of glycine and the positively charged BION surface,^54^ which could lead to a competitive displacement of rSpA molecules from the surface. Referring to this explanation, the salt addition may have prevented glycine from binding, e.g., due to the shielding of electrostatic interactions^55^ and, thus, could have counteracted the ligand leaching.

Based on the presented study, we chose sodium acetate as a suitable elution buffer because it revealed stable rSpA immobilization with simultaneously high IgG recovery. In further experiments, the increase in the ligand immobilization stability with increasing sodium acetate concentration (50–200 mM) was demonstrated at pH 2.4 (Figure 3B). Consistent with the above hypothesis of electrostatic shielding by salt, higher sodium acetate concentrations probably reduced the repulsion between positively charged BION (pI ∼ 6.0^34^) and ligands (pI ∼ 5.3^34^). However, around 13% less IgG was desorbed with 200 mM compared to 50 mM sodium acetate. As a compromise, we chose 100 mM sodium acetate at pH 2.8 as a robust buffer for the elution of IgG.

#### 3.2.3 Impact of BION@rSpA Concentration on Desorption Efficiency

**Figure 4:**
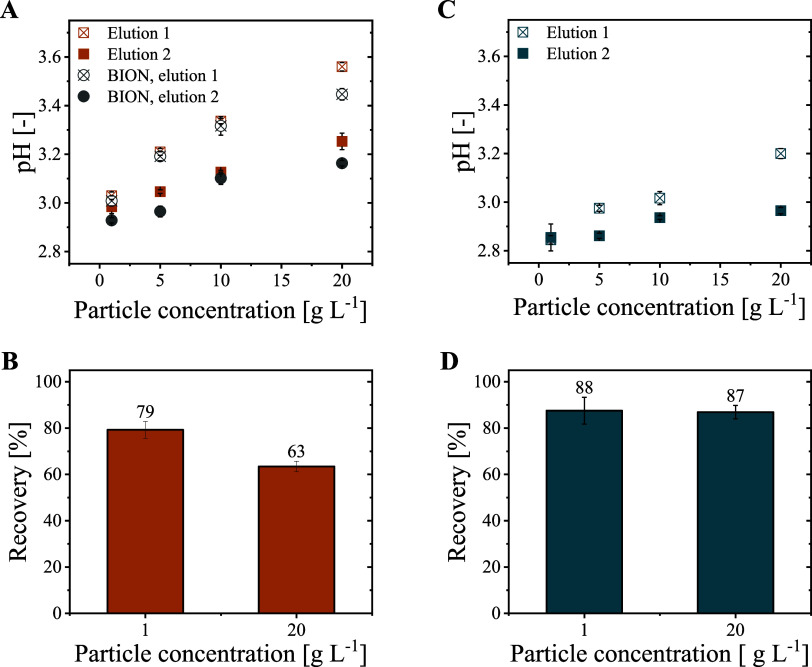
Impact of particle concentration on IgG desorption in two subsequent elution steps. (A) pH value of elution fractions recovered from 1 to 20 g L^–1^ BION@rSpA@IgG in 100 mM sodium acetate at pH 2.8. Also, simulated elution steps with BION (without proteins) are visualized. (B) IgG recovery from 1 and 20 g L^–1^ BION@rSpA@IgG in 100 mM sodium acetate at pH 2.8. (C) pH value of elution fractions recovered from 1 to 20 g L^–1^ BION@rSpA@IgG in 100 mM sodium acetate, 50 mM glycine at pH 2.8. (D) IgG recovery from 1 and 20 g L^–1^ BION@rSpA@IgG in 100 mM sodium acetate, 50 mM glycine at pH 2.8. In the experiments, IgG was bound to 5 g L^–1^ BION@rSpA before the BION@rSpA@IgG complex was diluted/concentrated to 1 or 20 g L^–1^ for elution. Two elution buffer exchange steps with 90% volume exchange were conducted.

Volume reduction is an important characteristic of a capture step in DSP. Magnetically concentrating the particles with adsorbed IgG (BION@rSpA@IgG) before desorption enables volume reduction and product concentration. In literature studies, magnetic protein separation is often only investigated at low particle concentrations between 1 and 5 g L^–1^.^34,39,49,56^ Differentiating from this, we analyzed the IgG desorption efficiency from up to 20 g L^–1^ particles in 100 mM sodium acetate buffer. Starting from pH 3.04 at 1 g L^–1^ particles, the pH in the eluates increased with the particle concentration to 3.56 at 20 g L^–1^ (Figure 4A). In a second elution buffer exchange step, the pH increase at 20 g L^–1^ particles was then reduced to 3.25. From 1 to 20 g L^–1^ particles, the IgG recovery decreased by over 15% (Figure 4B). Bare particles (without ligand and IgG) also led to a notable but weaker pH increase. For 20 g L^–1^ BION, a pH of 3.45 was measured in the first and 3.16 in the second elution step (Figure 4A).

The observed pH change resulted from a complex interplay between the iron oxides, buffer components, and proteins. To our knowledge, the pH effect has not yet been discussed concerning bioseparation applications. Oxides like the used iron oxide particles get protonated in an acidic buffer (below the isoelectric point) and deprotonated in an alkalic buffer (above the isoelectric point).^57−59^ Due to the protonation/deprotonation of the oxides, the bulk pH consequently increases/decreases. Two acidity constants (p*K*_a1_ ∼ 4.4; p*K*_a2_ ∼ 9.0 in KNO_3_ solution^60^) control the reactions. Another particle-specific parameter that influences proton interactions and the pH shift is the hydroxyl group density.^57^ The listed particle-specific parameters can be controlled, e.g., with suitable coatings, but they are set when working with BION. In addition to the particles, the buffer significantly impacts the bulk pH. The optimal buffering range of sodium acetate is around the p*K*_a_ value of 4.75 and not around 3 as applied in our case, which probably contributed to the ineffective buffering. Furthermore, acetate is predominantly protonated at around pH 3.0, and as the pH is near the p*K*_a_ value, a proton could be donated to the BION surface. With increasing particle concentration, more protons adsorb to the iron oxide surface, resulting in a stronger bulk pH increase. Immobilized rSpA ligands and present IgG molecules with pIs of ∼5.3^34^ and ∼9.0^61^ likely also underwent protonation in the acidic buffer, thus enhancing the pH increase caused by BION@rSpA@IgG compared to BION (Figure 4A). The pH increase was generally reduced with the second elution buffer exchange step, which probably resulted from already protonated surfaces and less dilution with entrapped buffer remaining from the previous processing at neutral pH.

As the predominating hydrophobic interactions between the B domain of Protein A and IgG decrease with lower pH,^8,9^ we assumed that the rise in pH contributed to the noticeable recovery loss at high particle concentrations (Figure 4B). Thus, we considered three strategies to enhance the buffering effectivity at low pH and to improve IgG elution: (1) a decrease of the initial buffer pH (e.g., 2.2–2.4) to reduce the resulting pH, (2) an increase of the buffering capacity, and (3) additives to enhance the buffering effect.

As discussed, the pH increase results from an equilibrium between large iron oxide particle surfaces and the surrounding liquid. Thus, until equilibrium is established, approach (1) forces very harsh elution conditions onto the IgG molecules. IgG can denature and subsequently aggregate at reduced pH values.^62^ Hence, a reduced pH is expected to impact the product quality negatively. Concerning approach (2), we observed that the pH at 20 g L^–1^ BION decreased with increasing sodium acetate concentration from 50 mM (pH 3.75) to 200 mM (pH 3.35). However, the IgG recoveries decreased with the buffer concentration, as noted in Section 3.2.2.

In approach (3), we investigated glycine as an additive to the 100 mM sodium acetate buffer. The p*K*_a_ value for the carboxyl group of glycine is 2.34, while the p*K*_a_ value of the amino group is 9.60.^63^ For example, at pH 3.0, the carboxy group is thus predominantly deprotonated, while the amino group is protonated. We expected the zwitterionic structure would benefit the acidic buffering efficiency of the elution buffer at increased particle concentrations. Experimental results confirmed the expected buffering improvement due to a 50 mM glycine additive (Figure 4C). With the additive, the pH increase at 20 g L^–1^ BION@rSpA was reduced by 0.36 to 3.20 in the first elution step (Figure 4A,C). It was further reduced to 2.97 in the second elution step, compared to 3.25 without the additive. The final pH of the eluates at 1 and 20 g L^–1^ differed only by 0.11 in the glycine-containing buffer (Figure 4C). Furthermore, the recovery at 20 g L^–1^ particles increased from 63 to 87% with the additive compared to pure sodium acetate (Figure 4B,D). No significant recovery loss was observed with glycine at increasing particle concentration (Figure 4D), probably due to the small difference between the final pH values (Figure 4C).

From the data, we concluded that the pH is the main contributor to efficient IgG elution from BION@rSpA@IgG, and no mechanical hindrance can be assumed in the studied particle concentration range. Furthermore, glycine has an intrinsic benefit on the desorption process. With SDS-PAGE analytics, we verified that the glycine additive did not lead to ligand leaching (Figure S.4) due to the stabilizing effect of sodium acetate, which we discussed in Section 3.2.2. Moreover, the BION@rSpA particles showed consistent IgG separation performance over three reuse cycles (Figure S.5), further confirming the ligand immobilization stability in the elution buffer system. Therefore, the 100 mM sodium acetate buffer with 50 mM glycine is suitable for the IgG purification process based on BION@rSpA.

#### 3.2.4 Impact of Agitation

During a magnetic antibody separation process, substantial mechanical stress acts on the particles and adsorbed proteins, e.g., during pumping or mixing for particle suspension.^64^ Exemplary, we here investigated IgG adsorption and desorption at varying shaking speeds (0, 500, 1000, 2000 rpm; Thermomixer C, Eppendorf). Furthermore, we assessed the effect of the shaking speeds on the IgG quality (aggregation) using DLS measurements.

**Figure 5:**
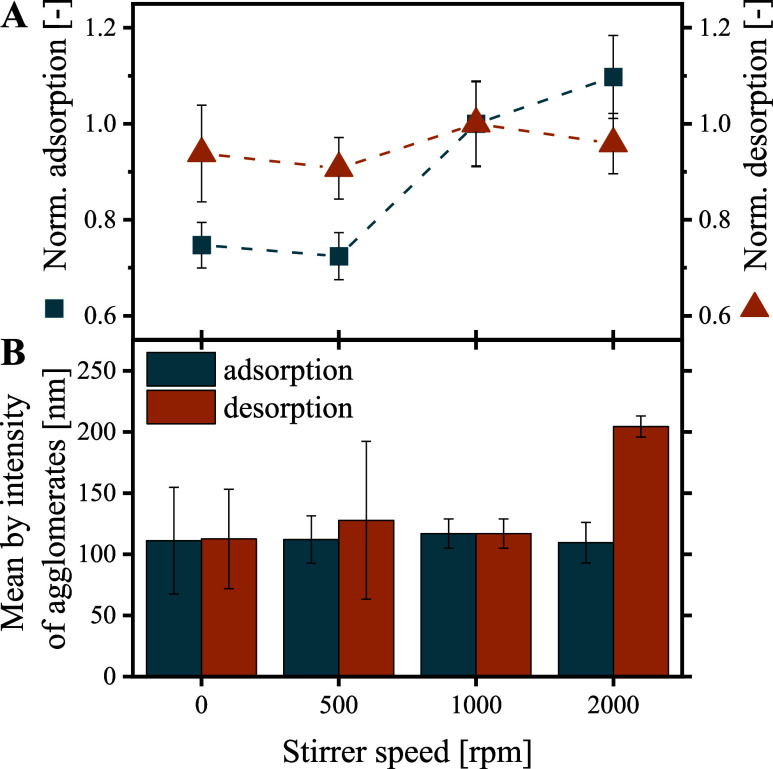
Impact of shaking speed on IgG adsorption and desorption onto/from 2 g L^–1^ BION@rSpA. (A) Relative IgG adsorption and desorption were plotted against the shaking speed. For the investigation of IgG desorption, IgG was adsorbed at 1000 rpm and desorbed at varying rpm parameters (Thermomixer C, Eppendorf). Values were normalized to the standard setting (1000 rpm) used within the publication. (B) Mean hydrodynamic diameter by intensity of agglomerates. Samples adsorbed at varying shaking speeds were desorbed at 1000 rpm, and samples desorbed at varying shaking speeds were adsorbed at 1000 rpm.

IgG adsorption to the BION@rSpA increased from 0 to 2000 rpm by about 40% (Figure 5A). Stirring influences the local IgG concentration around the particles and enhances the mass transport of IgG to the particles. Batch adsorption essentially depends on the shaking rate, as antibodies must come into contact with the ligand molecules to be able to bind.

In contrast to IgG binding, no noticeable impact of the shaking speed on IgG desorption was observed (Figure 5A). Thus, we concluded that affinity decreases between IgG and rSpA caused by the acidic elution buffer probably had a greater impact on the IgG desorption than mass transport effects.

DLS measurements of the neutralized eluates from the adsorption and desorption studies were used to assess IgG aggregation. Although this technique is generally only semiquantitative and does not give the accurate amount of monomers, dimers, and other high molecular weight species, it is suitable for estimating the aggregation behavior of proteins.^62,65,66^ The mean hydrodynamic diameter by intensity is very sensitive toward aggregation because the scattered light intensity strongly increases with rising diameter.^67^ Thus, we considered the hydrodynamic diameter of the aggregates (>100 nm) instead of monomeric IgG (12 nm^68^). Based on mean-by-intensity values, we could not observe an impact of the varied shaking speed during adsorption on the aggregation state (Figure 5B). However, DLS measurements of the samples desorbed at the highest investigated shaking speed of 2000 rpm revealed an increased mean hydrodynamic diameter of the aggregates (204.4 ± 8.7 nm) compared to 0 rpm (112.4 ± 40.7 nm) (Figure 5B). Consistently, the area by volume of the peak representing the monomer was 92.7 ± 1.3% for the sample eluted at 2000 rpm compared to 99.5 ± 0.4% at 0 rpm. In contrast to adsorption, the DLS measurements thus indicated a decrease in the monomer content and a simultaneous increase in aggregation resulting from vigorous shaking during acidic desorption.

Antibody aggregation upon neutralizing the acidic eluates in Protein A chromatography often results from protein denaturation in the acidic environment.^62^ Presumably, the non-native conformation of antibodies in acidic environments might reinforce a denaturation by mechanical stress. In contrast, the mainly native antibody conformation at neutral pH during the adsorption probably stabilized the monomeric protein molecule.^69^ Another possible explanation for the promoted aggregation at increased shaking speed could be the formation of IgG–ligand complexes resulting from the affinity increase between the two molecules after neutralization. However, this explanation is negligible because the ligand immobilization was stable, and no increased ligand leaching was detected in the IgG samples eluted at 2000 rpm (Figure S.4).

## 4 Conclusions

We investigated magnetic monoclonal antibody (mAb) separation based on bare iron oxide nanoparticles (BION) functionalized with an engineered Protein A ligand (rSpA) as an alternative to the conventional but limited Protein A chromatography. As commonly done, we used a Protein A-based ligand because of its high binding affinity to numerous commercially relevant IgG antibodies. However, our immobilization strategy of the (RH)_4_ tag-mediated, direct rSpA binding to the BION surface differed from common immobilization strategies that usually require elaborate chemical particle modifications (e.g., coatings).

Our direct immobilization approach simplifies particle synthesis and preserves the intrinsic properties of BION, which favor magnetic separation processes. BION@rSpA are small (8.4 nm) and have large specific surface areas, enabling high IgG binding capacities. In our studies, magnetophoretic attraction velocities surpassed those of coated particles, which allows accelerated processing. Beneficial for fast processing are also the shown rapid IgG adsorption (90% in 30 s) and desorption kinetics (90% in 2 min) onto/from BION@rSpA that exceed values of earlier studies. We explain the fast kinetics mainly with the efficient accessibility of the site-directed rSpA ligands on the BION surface. In our experiments, the IgG adsorption to BION@rSpA was highly selective and not impeded by impurity molecules in perfusion cell culture supernatant. Especially now, facing the increasing volumes from upstream processing (USP), the fast magnetic separation of IgG has great potential to counteract the throughput limitations of chromatography.

As a main challenge during our work with BION, we faced their strong pH buffering effect, which led to a significant pH increase upon concentrating the particles in an acidic 100 mM sodium acetate elution buffer. However, efficient acidic elution of IgG from rSpA can be reached by adding 50 mM glycine, which stabilizes the acidic pH and improves the recovery up to 87%, even at 20 g L^–1^ particles. Although this is already a high total particle concentration compared to earlier reported studies, we further plan to investigate higher particle concentrations up to 40–50 g L^–1^ to extrapolate this lab-scale study to the technical scale. It will also be interesting to see how the agitation in a rotor-stator high-gradient magnetic separator (RS-HGMS) impacts the elution efficiency.

Preparative magnetic bioseparation of mAbs is still in its infancy and presents only a small opponent to the established Protein A chromatography. However, magnetic separation is a promising technique in the ongoing process intensification movement, and studies like ours contribute to its progression. Our work suggests that magnetic separation using BION@rSpA has a high potential for efficient mAb DSP. A scale-up and transfer to RS-HGMS processing will be promising.

## Supporting Information Available

Phenanthroline assay used for quantification of iron oxide nanoparticles; magnetic field in space-and-time-resolved extinction profile (STEP) experiments; additional data on IgG adsorption kinetics; additional data on IgG elution in 100 mM sodium acetate + 50 mM glycine buffer (pH 2.8) at varying shaking speeds; reusability test of BION@rSpA over three cycles (PDF)

## Notes

The graphical abstract was created with BioRender.com.

## Abbreviations

**BCA**: bicinchoninic acid
**BION**: bare iron oxide nanoparticle
**cGMP**: current good manufacturing practice
**DLS**: dynamic light scattering
**DSP**: downstream processing
**DTT**: dithiothreitol
**HPLC**: high-performance liquid chromatography
**IgG**: immunoglobulin G
**IMAC**: immobilized metal affinity chromatography
**mAb**: monoclonal antibody
**PBS**: buffer consisting of 20 mM KH_2_PO_4_, 150 mM NaCl pH 7.4
**RS-HGMS**: rotor-stator high-gradient magnetic separator
**(RH)**_**4**_: peptide tag of four consecutive arginines and histidines
**rSpA**: engineered affinity ligand consisting of eight B-domains of Protein A and a fused (RH)_4_ peptide tag
SDS-PAGE: sodium-dodecyl sulfate-polyacrylamide gel electrophoresis
**STEP**: space- and time-resolved extinction profiles
**SQUID**: superconducting quantum interference device
**TBS**: buffer consisting of 20 mM Tris, 150 mM NaCl pH 7.0
**TEM**: transmission electron microscopy
**TEOS**: tetraethyl orthosilicate
**USP**: upstream processing
