# Supplementary material for: Direct Affinity Ligand Immobilization onto Bare Iron Oxide Nanoparticles Enables Efficient Magnetic Separation of Antibodies
Source: ACS Appl Bio Mater. 2024 May 13;7(6):3942–52. doi: 10.1021/acsabm.4c00280 (PMC11190986; doi:10.1021/acsabm.4c00280)
Supplement: Supplementary file 1 — mt4c00280_si_001.pdf [file mt4c00280_si_001.pdf]

Supporting Information for

Direct Affinity Ligand Immobilization onto Bare Iron Oxide Nanoparticles Enables Efficient Magnetic Separation of Antibodies

Ines Zimmermann [a], *Yasmin Kaveh-Baghbaderani* [a], *Friederike Eilts* [a], *Nadja Kohn* [a], *Paula Fraga-García* [a], *Sonja Berensmeier* [a,b,*]

[a] Chair of Bioseparation Engineering, TUM School of Engineering and Design, Technical University of Munich, Boltzmannstraße 15, 85748 Garching, Germany

[b] Munich Institute of Integrated Materials, Energy and Process Engineering, Technical University of Munich, Lichtenbergstraße 4a, 85748 Garching, Germany

[*] **Corresponding author:** Prof. Sonja Berensmeier, s.berensmeier@tum.de

S.1 Phenanthroline assay for quantification of iron oxide nanoparticles

The colorimetric phenanthroline assay is based on the formation of a chelate complex between Fe^{2+} ions and 1,10-Phenanthroline. Fe^{3+} ions must be reduced, e.g., in an ascorbic acid buffer. An acetate buffer at pH 4.5 stabilizes the complex. The required solutions are summarized in Table S.1.

Table S.1. Solutions required for the phenanthroline assay.

Buffer	Components	Volume or concentration
Acetate buffer pH 4.5	Acetic acid 96%<br>NaOH 1 M<br>Demineralized water	15 mL<br>130 mL<br>115 mL
Ascorbic acid	L(+)-ascorbic acid	10% (w/v)
Phenanthroline solution	1,10 Phenanthroline hydrochloride monohydrate	0.5% (w/v)
Fe^{2+} stock solution	Fe^{2+} (e.g $FeCl_2$)	0.1 mg mL^{-1}

Firstly, 40 µL of the iron oxide nanoparticle sample was mixed with 120 µL of concentrated HCl (37%). Afterward, 1840 µL demineralized water was added, and 20 µL from this mixture was added into a new reaction tube. Meanwhile, the calibration standard was prepared. Here, 0, 20, 40, 60, 100, 120, or 160 µL of the Fe^{2+} stock solution was added into a reaction tube. Next, 200 µL ascorbic acid and 800 µL acetate buffer were added to the samples and calibration standards and incubated for 5 min. Afterward, 100 µL of the Phenanthroline solution was added, and the tubes were incubated for 20 min. Lastly, the sample volumes were filled up to 2000 µL with demineralized water. For the measurement, 200 µL of the samples (triplicates) and calibration standards (duplicates) were pipetted into a 96-well microtiter plate, and the absorbance was measured at 510 nm using a microplate reader. The iron oxide concentration of particle samples was then calculated from the Fe^{2+} standard curve, considering the performed sample dilutions and the iron content in magnetite (Fe_3O_4).

S.2 Magnetic field in space-and time-resolved extinction profile (STEP) experiments

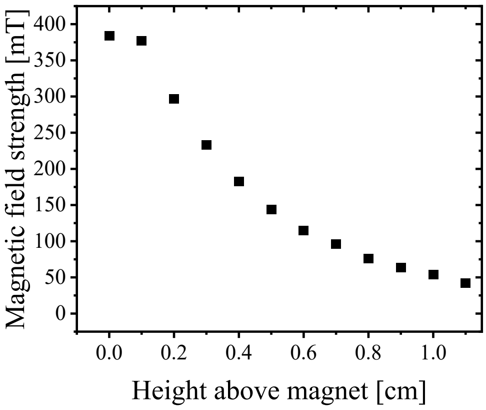

Figure S.1. The magnetic field strength above the five magnets (Webcraft GmbH, Germany) stacked in the used LUMiReader device (LUM GmbH, Germany) was determined with a Magnetic Meter PCE-MFM 3000 (PCE GmbH, Germany).

S.3 Additional data on IgG adsorption kinetics

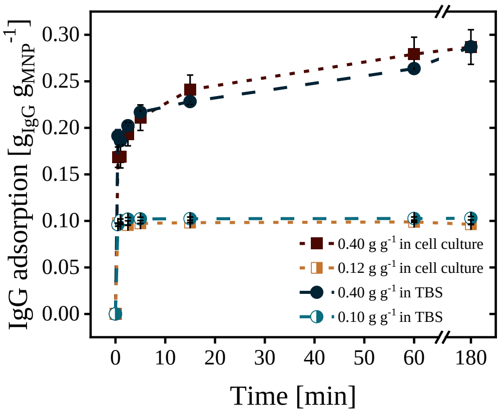

Figure S.2. IgG adsorption kinetics onto BION@rSpA. Here, the absolute values corresponding to Figure 3.A are plotted. Adsorption kinetics were studied with pure IgG in TBS buffer (20 mM Tris, 150 mM NaCl, pH 7.0) and IgG in cell culture supernatant at two initial IgG concentrations (0.10 – 0.12 g g^{-1} and 0.40 g g^{-1}).

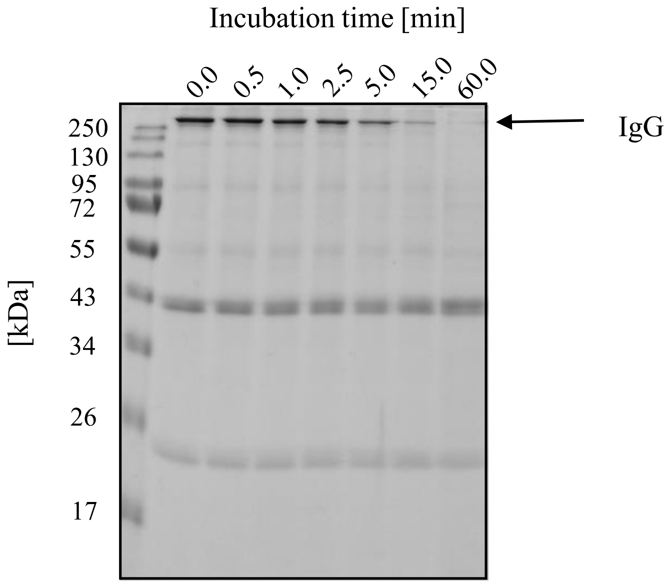

Figure S.3. Non-reducing SDS-PAGE analysis of the supenatants after incubation of cell culture supernatant (0.40 g L^{-1} IgG) with 3 g L^{-1} BION@rSpA over time (see section 3.2.1). Selective IgG adsorption to the particles can be seen, whereas impurity proteins remained in the supernatant. The antibody migrated higher than expected (150 kDa) in the gel, probaby because of its branched structure under the non-reducing conditions.

S.4 Additional data on IgG elution in 100 mM sodium acetate + 50 mM glycine buffer (pH 2.8) at varying shaking speeds

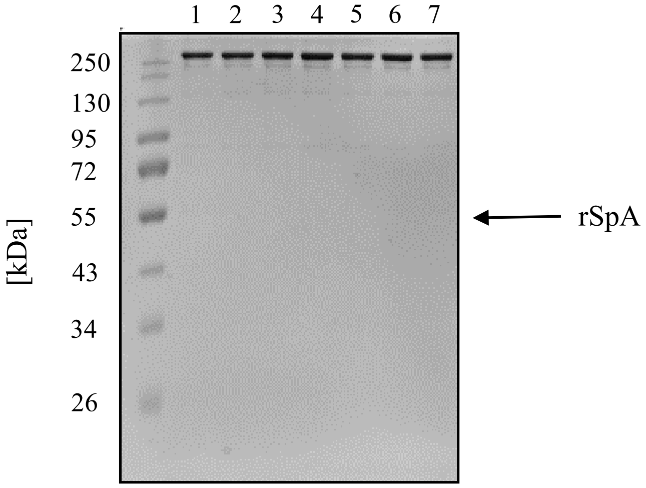

Figure S.4. Non-reducing SDS-PAGE analysis of IgG samples eluted in 100 mM sodium acetate, 50 mM glycine (pH 2.8) at varying stirring speeds. Lane (1) adsorption at 0 rpm, elution at 1000 rpm; lane (2) adsorption at 500 rpm, elution at 1000 rpm; lane (3) adsorption at 1000 rpm, elution at 1000 rpm; lane (4) adsorption at 2000 rpm, elution at 1000 rpm; lane (5) adsorption at 1000 rpm, elution at 0 rpm; lane (6) adsorption at 1000 rpm, elution at 500 rpm; lane (7) adsorption at 1000 rpm, elution at 2000 rpm. In no sample, rSpA leaching into the eluates was detected with the software ImageQuant TL (Cytiva, USA). The antibody migrated higher than expected (150 kDa) in the gel, probaby because of its branched structure under the non-reducing conditions.

S.5 Reusability test of BION@rSpA over three cycles

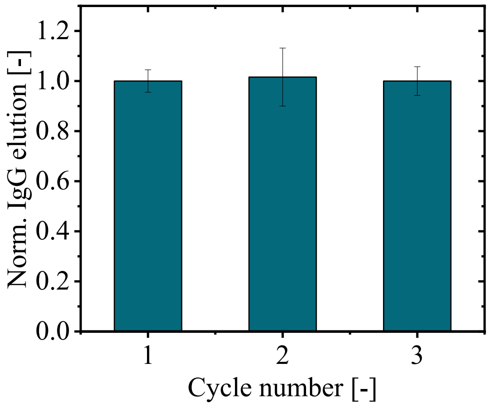

Figure S.5. Reusability of BION@rSpA (2 g L^{-1}) over three cycles. Shown are the eluted IgG amounts normalized to the elution in the first cycle. Each use cycle consisted of IgG adsorption onto BION@rSpA (0.1 g g^{-1}) in TBS buffer (20 mM Tris, 150 mM NaCl, pH 7.0), washing in TBS (2x) and deionized water (1x), and elution in 100 mM sodium acetate, 50 mM glycine at pH 2.8. Between the cycles, the particles were additionally incubated in the elution buffer for particle cleaning.
